# Serum high expression of miR-214 and miR-135b as novel predictor for myeloma bone disease development and prognosis

**DOI:** 10.18632/oncotarget.7319

**Published:** 2016-02-11

**Authors:** Mu Hao, Meirong Zang, Lei Zhao, Shuhui Deng, Yan Xu, Fang Qi, Gang An, Yu Qin, Weiwei Sui, Fei Li, Wenjuan Yang, Zengjun Li, Shuhua Yi, Dehui Zou, Fenghuang Zhan, Lugui Qiu

**Affiliations:** ^1^ State Key Laboratory of Experimental Hematology, Institute of Hematology and Blood Diseases Hospital, Chinese Academy of Medical Science and Peking Union Medical College, Tianjin 300020, China; ^2^ Division of Hematology, Oncology, and Blood and Marrow Transplantation, Department of Internal Medicine, University of Iowa, Iowa City, IA 52242, USA; ^3^ Department of Molecular Physiology and Biophysics, Holden Comprehensive Cancer Center, University of Iowa Carver College of Medicine, Iowa City, IA 52242, USA

**Keywords:** serum miRNAs, bone disease, multiple myeloma, biomarkers

## Abstract

Multiple myeloma (MM) originates from malignant plasma cells, leading to multiple destructive lytic bone lesions that occur in more than 80% of MM patients. MicroRNAs have been reported to be involved in development of bone lesions in MM. However, the circulating microRNA as diagnostic and prognostic biomarkers for bone lesions has not been elucidated yet. In this study, we identified differentially expressed miRNAs that are potentially involved in myeloma-related bone disease in serum of MM patients. MiR-214 and miR-135b was shown to be increased in serum of MM patients with bone lesions. Serum level of miR-214 and miR-135b was highly correlated with the severity of lytic bone lesions and demonstrated as a diagnostic tool for identifying bone diseases based on results of a receiver operating characteristic analysis (ROC). In addition, patients with high levels of serum miR-214 had a dismal survival with significantly shortened progression free survival (PFS) and overall survival (OS). Interestingly, bisphosphonates treatment significantly extended PFS and OS in patients with higher level of miR-214 comparing to patients without bisphosphonates treatment. Taken together, our findings revealed the significance of circulating miR-214 and miR-135b levels in detection of bone disease and in prediction of prognosis of patients with multiple myeloma, suggesting its potential clinical applications. The result of this study also set the foundation for searching more circulating miRNA as biomarker for tumor bone lesions.

## INTRODUCTION

Multiple myeloma (MM) originates from clonal expansion of malignant plasma cells in bone marrow, leading to multiple destructive lytic bone lesions at the time of diagnosis [1]. The procedures that are widely used in nowadays in the diagnosis and monitoring of myeloma-related bone disease include conventional radiography, computed tomography (CT), magnetic resonance imaging (MRI) and positron emission tomography/CT (PET/CT) [3]. In addition, biochemical markers of bone turnover, such as Alkaline Phosphatase (ALP), serum calcium, receptor activator of nuclear factor-kappa B ligand (RANKL), osteoprotegerin, osteopontin, provide information on bone dynamics that in turn may reflect disease activity in bone [2]. However, identification of more easier, non-invasive and accurate biomarkers for myeloma-related bone diseases, even in the absence of lytic lesions on conventional radiography and definition of a myeloma-related fracture still are challenges for physicians.

MicroRNAs (miRNAs) are an abundant class of regulatory noncoding single-strand RNA molecules approximately 20–23 nucleotides long. In general, miRNAs bind with imperfect complementarity to the 3′-untranslated region (3′-UTR) of a specific target mRNA to promote its degradation and/or inhibit its translation [4]. Many miRNAs display regulatory roles in almost all major biological processes, including cell motility, differentiation, proliferation and apoptosis [3–5]. Dysregulation of miRNAs in MM cells and bone marrow microenvironment has important impacts on the initiation and progression of myeloma bone disease [6, 7]. Recently, it was reported that serum and plasma of MM patients contained sufficiently stable miRNA signatures. Circulating miRNAs could be the minimally invasive way of acquiring samples from body fluid and be measured accurately. Compared to conventional diagnostic parameters, the circulating miRNA profile is appropriate for the diagnosis of bone disease and estimates patient progression and therapeutic outcome with higher specificity and sensitivity. However, the diagnostic and prognostic values of circulating miRNA in bone diseases, including myeloma bone disease have not been evaluated. In the present study, we mainly focus on the potential clinical significance of circulating miRNAs as diagnostic, prognostic, and predictive biomarkers for myeloma bone disease.

## RESULTS

### Patient's characteristics

A total of 152 serum samples including 108 newly diagnosed symptomatic MM patients and 44 healthy donors (HD) were enrolled in this study. The median age of the patients was 55 years old (range 33–83 yr). Among the newly diagnosed patients, 55 received bortezomib-based treatment (arm A) and 53 received thalidomide-based treatment (arm B). 104 of them have documented whole body X-ray scanning data [8–10] with a median follow-up time of 13.5 months upon diagnosis. And 59 patients received bisphosphonate treatment. There was no significant difference in clinical and cytogenetic characteristics between arm A and arm B. The clinical characteristics of these 108 newly diagnosed patients are shown in Table 1.

**Table 1:** Patients' and healthy donors' base-line characteristics

	Healthy donors		MM patients	
Charateristic	(*n* = 44)	Treatment arm A (*n* = 64) n/N (%)	Treatment arm B (*n* = 44) n/N (%)	*p* value
Gender				
Male	25	44 (68.8)	28 (63.6)	NA
Female	19	20 (31.2)	16 (36.4)	NA
Age (years)	55	58	59	NA
Range	45–78	33–77	37–83	NA
ISS stage				0.394
I	NA	14/64 (21.9)	9/44 (20.5)	
II	NA	21/64 (32.8)	14/44 (31.8)	
III	NA	29/64 (45.3)	21/44 (47.7)	
β2-microglobulin				0.167
< 5.5 mg/dL	NA	33/64 (51.6)	21/44 (47.7)	
≥ 5.5 mg/dL	NA	31/64 (48.4)	23/44 (52.3)	
Durie-Salmon stage, %				0.356
I–II	NA	11/64 (17.2)	5/44 (11.4)	
III	NA	53/64 (82.8)	39/44 (88.6)	
Bone disease stage				0.070
0	NA	9/64 (14.1)	5/44 (11.4)	
1	NA	14/64 (21.9)	10/44 (22.7)	
2	NA	8/64 (12.5)	7/44 (15.9)	
3	NA	28/64 (43.7)	16/44 (36.4)	
4	NA	5/64 (7.8)	6/44 (13.6)	

ISS: International Staging Systerm; NA: not applicable.

### Dysregulation of serum miRNAs in MM patients

**Figure 1:**
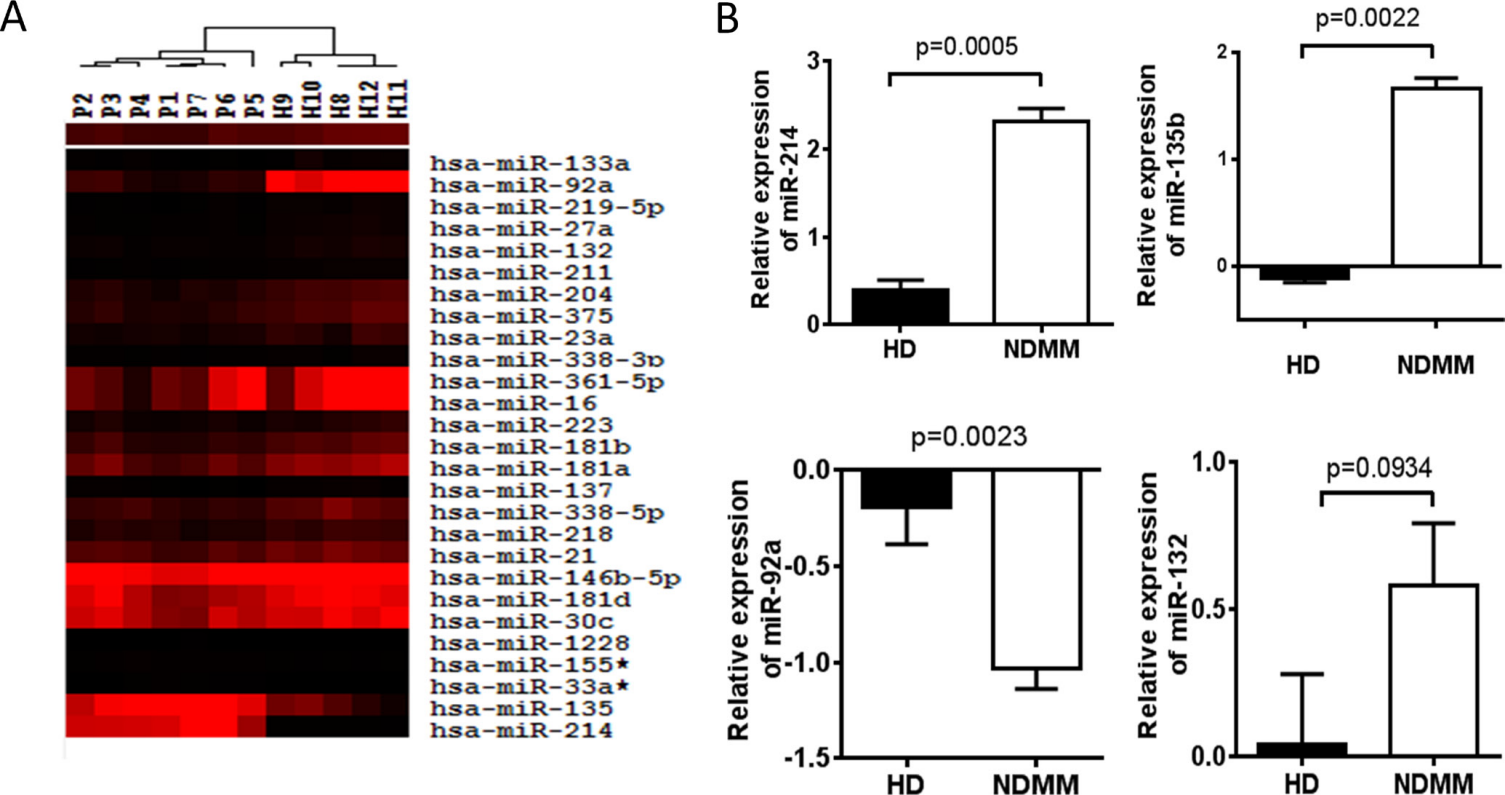
Dysregulation of serum miRNAs in MM patients (**A**) Hierarchical clustering analysis of serum miRNA expression associated with bone disease in myeloma patients. The expression of miRNA is hierarchically clustered on the y-axis, and serum samples of MM patients or healthy control are hierarchically clustered on the x-axis. The legend indicates the miRNA represented in the corresponding row. Relative miRNA expression is depicted according to the color scale as shown. Red and black indicates up-regulation and down-regulation, respectively. Numbers with P indicate MM patient samples; numbers with H indicate healthy control samples. (**B**) Validation of candidate miRNAs using RT-qPCR. Relative expression of 4 miRNAs on a large cohort of 108 newly diagnosed MM patients (NDMM) and 44 HD samples were measured by RT-qPCR. MiRNA expression for each sample was normalized to the internal control miR-423–5p which is stably expressed in serum of MM patients and calculated with 2^−ΔΔct^. Statistical significance was determined by a Student's *t*-test. Significance was defined as *p* < 0.05.

To evaluate levels of miRNAs in serum of MM patients and healthy donors, serum samples were collected randomly from seven newly diagnosed MM patients with different stage of bone disease and five healthy donors. MiRNA profiles were performed using the miRCURY^™^ LNA Array which probes 1891 miRNAs. The array identified that twenty-seven miRNAs which were involved in regulation of bone formation and resorption were significantly dysregulated (the cut-off of fold change is ≥ 1.5, *p* < 0.05) between MM patients and healthy donors. Among them, four (14.3%) miRNAs were up-regulated and twenty-three (85.7%) were down-regulated (Table 2 and Figure 1A). miR-214 (fold change of 4.8), miR-135b (fold change of 3.6), miR-132 (fold change of 0.43) and miR-92a (fold change of 0.49) were of particular interest due to their critical role in regulating differentiation of osteoclast and osteoblast as reported by the literatures [11–13].

**Table 2:** Differentially expressed miRNAs between MM patients and HDs

	Fold Change	*P*-value
Name	Pt vs HD	Pt vs HD
hsa-miR-219-5p	0.300533	0.000274
hsa-miR-27a	0.321873	1.19E-05
hsa-miR-133a	0.345897	0.029937
hsa-miR-211	0.418319	2.38E-05
hsa-miR-132	0.433217	0.000399
hsa-miR-204	0.445862	6.18E-05
hsa-miR-23a	0.450964	0.002324
hsa-miR-361-5p	0.467681	0.035313
hsa-miR-16	0.467681	0.035313
hsa-miR-375	0.483455	0.000273
hsa-miR-92a	0.493495	0.006058
hsa-miR-137	0.528215	0.015496
hsa-miR-223	0.529003	0.015473
hsa-miR-338-5p	0.54475	0.000701
hsa-miR-181b	0.552612	0.001189
hsa-miR-338-3p	0.558305	0.025214
hsa-miR-181a	0.577981	0.000687
hsa-miR-1228	0.60316	0.03915
hsa-miR-218	0.604278	0.001185
hsa-miR-21	0.713682	0.001676
hsa-miR-146b-5p	0.733103	0.000486
hsa-miR-181d	0.749033	0.016606
hsa-miR-30c	0.78512	0.028978
hsa-miR-155*	1.745892	0.000257
hsa-miR-33a*	3.253112	0.003044
hsa-miR-135b	3.550036	0.000183
hsa-miR-214	4.820454	0.000596

Pt: MM patients; HD: healthy donor of serum.

### MiR-214 and miR-135b level is highly correlated with bone disease of MM patients

miRNAs expression in serum was further validated in a large cohort of 108 newly diagnosed MM patients and 44 HDs by the miRNA-specific RT-qPCR assay with miR-423–5p used as an internal control [14, 15]. The results confirmed that the level of miR-214 (2.34 vs. 0.23, *p* = 0.0005) and miR-135b (1.83 vs. −0.18, *p* = 0.0022) was significantly increased in MM patients compared to HDs, while the level of miR-92a (−0.98 vs. −0.47, *p* = 0.0023) was significantly decreased in patients. However, we did not find that miR-132 was obviously altered between normal and patients serum (Figure 1B).

**Figure 2:**
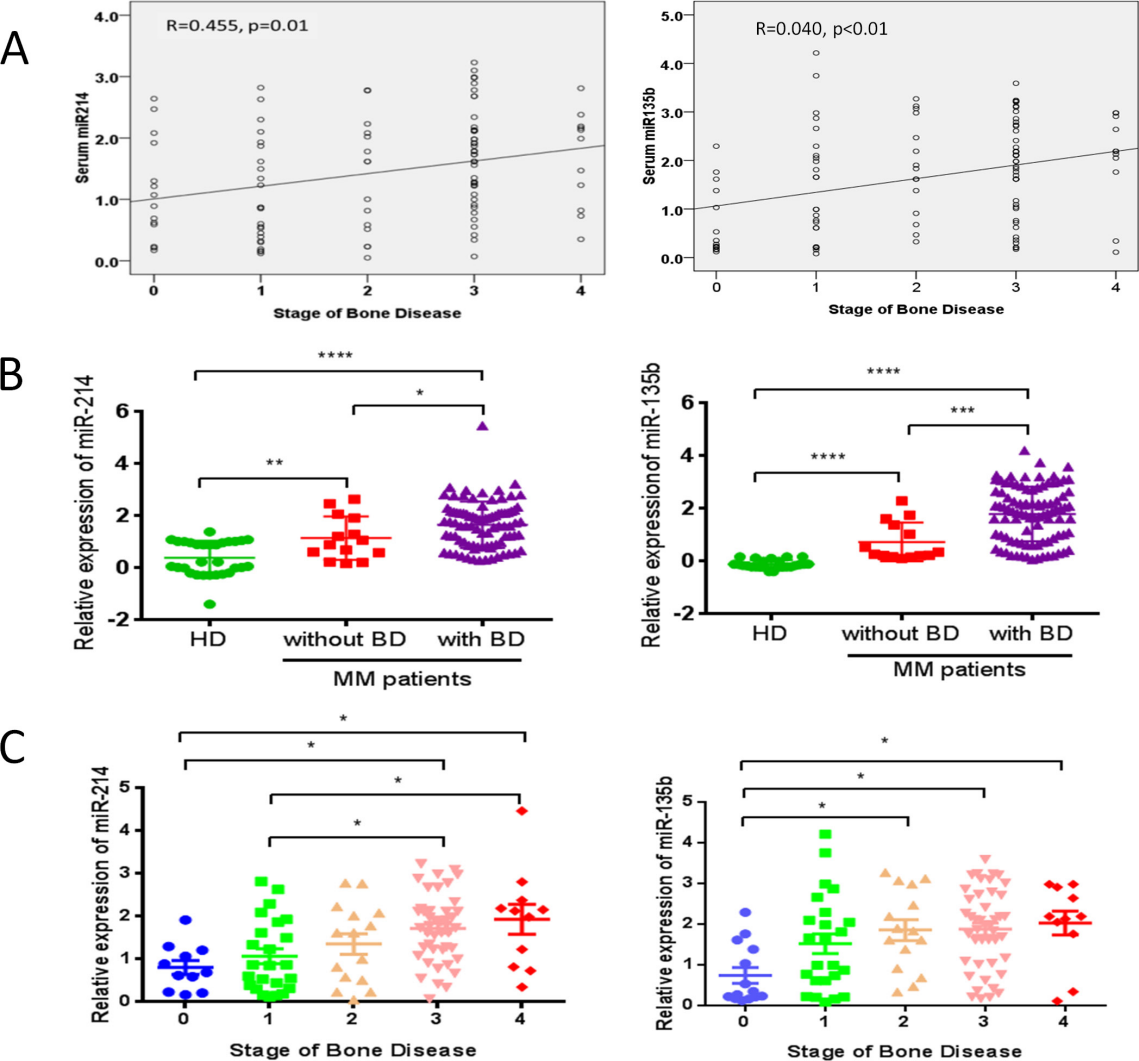
miR-214 and miR-135b levels were highly correlated with bone disease of MM patients (**A**) Correlation analysis of serum miR-214 and miR-135b with stage of bone disease in MM patients by Pearson-moment correlation coefficient calculations. (**B**) Expression of serum miR-214 and miR-135b were measured via using RT-qPCR in healthy donors (HD) and MM patients with (*n* = 94) or without (*n* = 14) bone disease. (**C**) Expression of miR-214 and miR-135b was up-regulated according to the stages of bone disease (stage0, *n* = 14; stage1, *n* = 24; stage2, *n* = 15; stage3, *n* = 44; stage4, *n* = 11). For (B) and (C), statistical analysis was performed using *one-way* ANOVA followed by Tukey multiple comparisons (*p < 0.05, **p < 0.01, ***p < 0.001).

We then investigated the correlation of serum levels of miR-214, miR-135b and miR-92a with severity of bone lytic lesions in 104 newly diagnosed MM patients via Pearson-moment correlation coefficient calculations. Grading of lytic bone lesions was determined based on X-ray radiographic data as previously described [8, 9, 16]. The results indicated that levels of circulating miR-214 (*r* = 0.455, *p* = 0.01) and miR-135b (*r* = 0.404, *p* < 0.01) were highly correlated with bone lytic lesions in this cohort of patients (Figure 2A). However, our data didn't show a significant correlation of miR-92a serum levels with bone disease in these patients (data not shown). Further comparison of serum levels of miR-214 and miR-135b in MM patients with or without lytic bone lesions revealed that the levels of miR-214 and miR-135b in patients with bone disease were significantly higher than those without bone disease (both *p* < 0.0001, Figure 2B). Moreover, patients with more advanced bone lesions have significantly higher levels of serum miR-214 and miR-135b (Figure 2C, *p* < 0.05). These results strongly suggested that serum miR-214 and miR-135b levels were highly correlated with bone disease of MM patients.

### MiR-214 and miR-135b offer a powerful diagnostic tool for identification of bone disease related to myeloma

**Figure 3:**
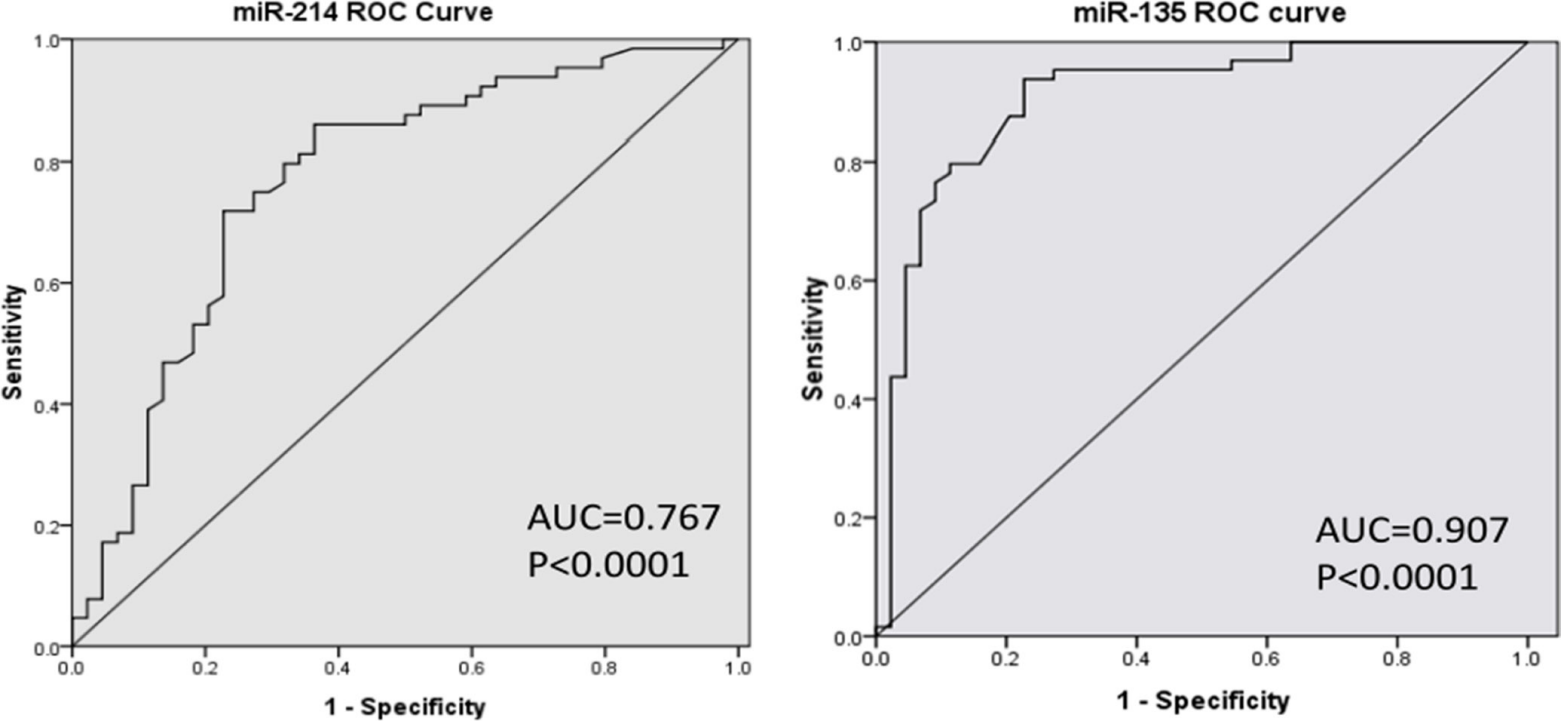
MiR-214 and miR-135b offer a powerful diagnostic tool in identification of lytic bone lesion in MM patients ROC analysis showed that the area under the curve (AUC) of miR-214 was 0.767 (*p* < 0.001), while the AUC of miR-135b was 0.907 (*p* < 0.001). Serum miR-135b was a more powerful diagnostic tool than miR-214 in distinguishing myeloma patients with or without bone disease.

Next, we investigated the ability of miR-214 and miR-135b to distinguish MM patients with or without bone disease using the ROC analysis. The results revealed that serum levels of miR-214 and miR-135b both could be used to distinguish MM patient with bone disease from those without bone lesions. The area under the curve (AUC) of miR-214 was 0.767 with 97% sensitivity and 86% specificity. Furthermore, the serum level of miR-135b was a more powerful diagnostic tool in identification of myeloma bone disease with an AUC of 0.907, sensitivity of 100% and specificity of 73% (*p* < 0.001, Figure 3). Taken together, these data demonstrated that patients with bone lesions have higher levels of circulating miR-214 and miR-135b. The level of these two miRNAs is positively correlated with the severity of bone disease. High level of miR-214 and miR-135b could be used as a diagnostic biomarker for distinguishing bone disease and for evaluating the severity of bone disease in MM.

### High miR-214 level is a powerful predictor for poor prognosis of myeloma

**Figure 4:**
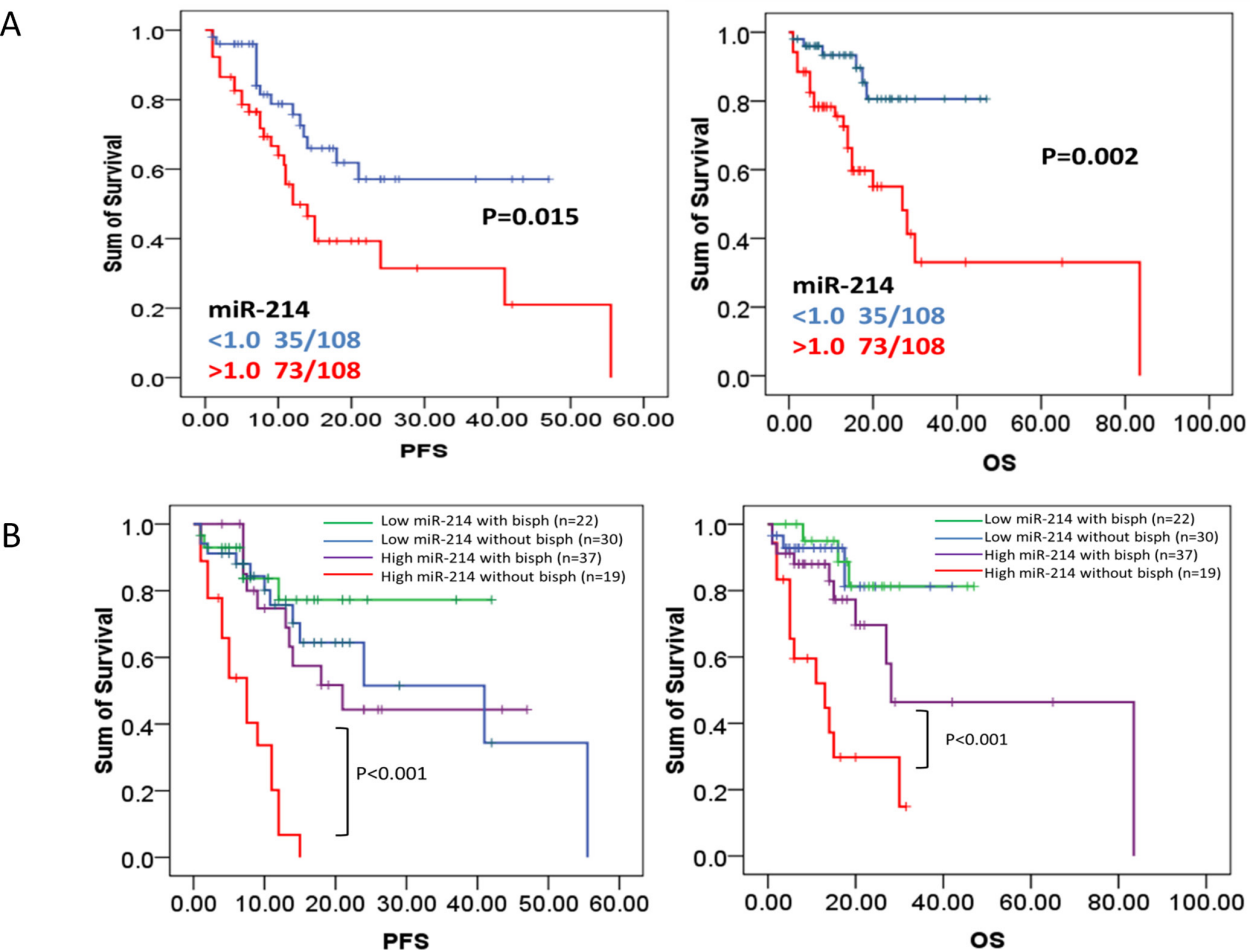
High level of miR-214 predicts poor prognosis in newly diagnosed myeloma patients (**A**) PFS and OS were investigated for MM patients according to expression of serum miR-214 in 108 MM patients. Survival analysis was performed via Kaplan–Meier survival analysis, with differences between curves analyzed via a log-rank test. Significance was defined as *p* < 0.05. (**B**) Bisphosphonates treatment significantly extended survival of patients with high serum miR-214 levels. PFS and OS were evaluated for MM patients having high serum level of miR-214 with or without bisphosphonates treatment. Survival analysis was performed using Kaplan–Meier survival analysis, with the differences between curves analyzed via a log-rank test. Significance was defined as *p* < 0.05.

We have shown that circulating miR-214 and miR-135b can serve as a diagnostic tool in identifying bone disease of MM patients. We then queried the impact of miRNA expression on survival in newly diagnosed MM patients. The 108 newly diagnosed patients with complete clinical data were grouped into either a high or low miR-214/miR-135b group with the Youden's Index used to identify the optimal cutoff point for miRNAs expression grouping. Our finding discovered that circulating level of miR-214 exhibited a negative impact on patients' survival. Patients with up-regulated miR-214 had significantly shortened progression free survival (PFS 8.0 v.s. 22.0, *p* = 0.015) and overall survival (OS 15.0 v.s. 28.0 months, *p* = 0.002), as Figure 4A showed. However, we failed to find any prognostic value of miR-135b in newly diagnosed MM patients (data not shown).

### Bisphosphonates treatment benefits patients with higher serum miR-214 level

We have shown that high level of serum miR-214 can serve as an indicator of bone disease and as a predictor of poor outcome in MM patients. We further investigated whether levels of miR-214 factored in patients' response to different therapeutic regimens. As we expected, bisphosphonates treatment significantly extended PFS and OS of MM patients with higher level of miR-214 (defined by cutoff > 1.0). The median PFS was significantly prolonged to 22 months compared to 8 months in patients without bisphosphonates treatment (*p* < 0.001). The median OS was significantly prolonged to 28 months compared to 15 months in patients without treatment (*p* < 0.001, Figure 3B). These results suggested that bisphosphonates treatment benefits the patients with higher serum miR-214 level by significantly extending their survival. Altogether, our data indicated that bisphosphonates is beneficial to patients with up-regulation of serum miR-214 even in the early stage of disease when bone lesion cannot be detected. However, our data did not show that bortezomib-based therapy could extend either the PFS or overall survival of patients with high level of miR-214 (Supplementary Figure1).

## DISCUSSION

Biomarkers are essential tools in clinical research and practice. Useful biomarkers must combine good measurability, validated association with biological processes or outcomes, and should support clinical decision making if used in clinical practices. MiRNAs are small, noncoding RNAs that repress gene expression by primarily interacting with the 3′ untranslated region (UTR) of their target mRNA transcripts. Development of circulating miRNAs as biomarkers is a rapidly growing research area in the last decade. In association with other biomarkers, changes in levels of circulating miRNAs offer the potential for a highly sensitive and specific tumor detection and classification system. Circulating miRNAs are a minimal or non-invasive source of biomarkers as they are contained in serum and plasma samples, cerebrospinal fluid, and saliva allowing minimal-invasive detection and therefore a broad applicability in clinics and research studies. This is especially advantageous for diseases affecting tissues that are not easily accessible for biopsies such as bone. In addition, miRNAs are robust for analysis, because, once collected, circulating miRNAs exhibit remarkable stability in body fluids even after prolonged exposure (up to 24 h) to room temperature [17]. This makes circulating miRNAs become advantageous in clinical routine and in an outpatient setting. Others and our previous study described that serum miRNA profiles could be used as a novel predictor for disease progression and prognosis in multiple myeloma [15, 18, 19]. However, there were few investigations of circulating miRNAs as biomarkers for myeloma-related bone disease.

A number of studies had identified that miRNAs are stably present in serum and target critical genes in osteoclast and osteoblast differentiation and play a notable role in osteoclastogenesis in tumor [12, 13, 20–22]. Our previous study has identified that the level of miRNAs in serum is dysregulated in myeloma patients and associated with disease progression and prognosis. In the present study, the diagnostic and prognostic value of serum miRNAs which are correlated with bone homeostasis in myeloma bone disease was elucidated.

In the present study, microarray data indicated that twenty-seven miRNAs involved in bone homeostasis were significantly dysregulated (23 decreased and 4 increased) in myeloma patients compared with healthy donors (Figure 1 and Table 2). In a large cohort of 108 newly diagnosed MM patient samples and 44 healthy controls, RT-qPCR consistently confirmed that serum level of miR-214 and miR-135b was significantly increased in MM patients, while miR-92a was notably decreased. Correlation analysis indicated that the level of miR-214 (*r* = 0.455, *p* = 0.01), miR-135b (*r* = 0.404, *p* < 0.01) but not miR-92 were highly correlated with bone lesions on radiography scanning as Figure 2A showed. Moreover, miR-214 and miR-135b level was further increased in myeloma patients with bone lytic lesions compared with those without bone lesions (Figure 2B). Clinical characteristic analysis demonstrated that miR-214 and miR-135b level was significantly increased according to the severity of myeloma bone disease. The level of miR-214 and miR-135b was significantly increased in patients with more severe lytic lesion compared to those with less lytic lesions (Figure 2C). Altogether, these data indicated that the level of miR-214 and miR-135b was increased in myeloma patients. Level of miR-214 and miR-135b highly correlated with myeloma bone disease. The patients with extensive bone lesions have increased miR-214 and miR-135b. Furthermore, ROC analysis demonstrated that serum miR-214 and miR-135b can be used to discriminate myeloma patients with or without bone lytic lesions. MiR-135b was even a more powerful diagnostic tool in distinguishing MM patients with or without bone lytic lesions with an AUC value of 0.907 and 100% sensitivity and 73% specificity. Survival analysis indicated that patients with high level of miR-214 exhibited a dismal survival with shortened PFS and OS. However, level of miR-135b did not show correlation with PFS and OS of MM patients. Furthermore, bisphosphonates treatment benefits patients with higher serum miR-214 level by significantly extending their survival.

Activation of osteoclast and suppression of osteoblast are involved in myeloma bone disease. A number of studies indicated that miRNA was involved in regulation of osteoclast and osteoblast differentiation [11, 13, 21–23]. miR-214 plays critical roles both in osteoclast activation [24] and osteoblast suppression [7, 25] by targeting different mRNA transcripts. miR-214 is up-regulated during osteoclastogenesis from bone marrow monocytes (BMMs), which is inducted by macrophage colony stimulating factor (M-CSF) and receptor activator of nuclear factor-κB ligand (RANKL). Moreover, miR-214 inhibits osteoblast function by targeting ATF4, a transcription factor that promotes the expression of osteoblast-specific genes, such as osteocalcin. Additionally, miRNA-214 binds to Osterix and suppresses osteoblast differentiation. For miR-135b, Schaap-Oziemlak *et al*. reported that miR-135b down-regulation is functionally important for full osteogenic differentiation and mineralization of human unrestricted somatic stem cells [13]. miR-135b has been shown to impair breast cancer progression and metastatic osteolytic bone disease via targeting Runx2 [26]. Xu *et al*. reported that up-regulation of miR-135b was involved in compromised osteogenic differentiation of mesenchymal stem cells derived from MM Patients [27]. Therefore, miR-214 and miR-135b play critical roles in tumor associated bone disease development. This may explain that dysregulated miR-214 and miR-135b were highly linked with bone disease in myeloma patients and could be used to distinguish patients with or without bone disease.

More than 80% myeloma patients have osteolysis, leading to fractures, severe bone pain, spinal cord compression and greatly compromising patient quality of life and resulting in mortality. We then wondered the prognostic value of circulating miR-214 and miR-135b level in myeloma patients. Survival analysis revealed that patients with higher serum level of miR-214 (cutoff > 1.0) had a significantly shortened PFS (12.0 months *v.s*. NR, *p* = 0.015) and OS (28.0 month *v.s*. NR, *p* = 0.002, Figure 3A). However, we did not find that miR-135b level was correlated the survival of myeloma patients. Interestingly, clinical characteristic analysis revealed that the patients with higher level of serum miR-214 had more benefit from the treatment of bisphosphonates regimens. Bisphosphonates therapy significantly extended the survival of patients with higher level of serum miR-214 (8.0 *v.s*. 22.0 months for PFS, 15.0 *v.s*. 28.0 months for OS, both *p* < 0.02). These results strongly suggested that bisphosphonates was an effective treatment for patients with high level of circulating miR-214, and further supported that the validity of up-regulated level of serum miR-214 could be used as an important indicator for bisphosphonates treatment. The studies showed [28, 29] that bisphosphonate treatment significantly suppressed osteoclast differentiation and inhibited myeloma cell proliferation. Bisphosphonate treatment not only decreases tumor burden, but also inhibits myeloma bone disease development. Our study did not find significant difference in survival of patients with high level of miR-214 patients treated with or without bortezomib. These results meant that the level of miR-214 was much more associated with myeloma bone destruction rather than tumor burden.

Unfortunately, limited information is available describing the function of miR-214, although it is found to be dysregulated in many cancers [30]. Misiewicz-Krzeminska *et al*. showed that restoration of miRNA-214 expression reduced growth of myeloma cells through positive regulation of p53 and inhibition of DNA replication [31]. Our results closely mirror the results of Misiewicz-Krzeminska's study. However, we go further to show that MM patients with a high miR-214 serum expression have poor outcome, suggesting that miR-214 may target genes essential to cellular repression. Interestingly, some dysregulated circulating miRNAs are different from their counterparts detected within myeloma cells as Yoshizawa *et al*. and our previous study reported that miR-92a is down-regulated in plasma, but it is part of the up-regulated miR-17–92 cluster in MM cells [15, 32, 33]. This trend is further supported by present study. miR-214 was up-regulated in serum of MM patients but down-regulated in MM cells (data not shown).

It has been shown that loci for miRNAs are frequently located in fragile, cancer-associated genomic areas [15, 34]. Regarding the chromosomal loci, one possible mechanism accounting for up-regulation of serum miR-214 and miR-135b is a gain of the chromosome region 1q in myeloma cells. Extra copies of chromosome 1q are among the most commonly reported genetic abnormalities in multiple myeloma (MM) [35]. An *et al*. identified that patients with cytogenetic abnormality 1q gain are considered as high-risk MM with poor outcome [36]. Of particular interests, studies by Jindra *et al.*, Yin *et al.* and Yang *et al.* have identified miR-214 as a key mediator of PTEN expression. Their data suggest that overexpression of miR-214 results in the down-regulation of PTEN protein and activation of Akt pathway [37–39]. This could be potential explanation to the fact that patients with high miR-214 expression had significantly inferior outcome in our study. In sum, these findings suggest that overexpression of miR-214 induces cell survival through suppression of PTEN and activation of PI3K/AKT pathway. This could be related to that high miR-214 expression leading to increase in cell proliferation and poor outcome in patients with myeloma.

In summary, our data offer novel insights into miR-214 and miR-135b in MM. We demonstrate that the expression of miRNAs in serum of MM patients varies significantly. These results suggest that miR-214 and miR-135b may serve as biomarkers for detection of myeloma bone disease. Interestingly, miR-214 may serve as an important prognostic marker for MM with bone disease and for initiation of bisphosphonate treatment. The results of our study show the importance of miR-214 and miR-135b serum expression in myeloma bone disease diagnosis and prognosis, suggesting that clinical applications are appropriate. Finally, this study set the foundation for studying serum miRNA as biomarkers for tumor-related bone lesions.

## MATERIALS AND METHODS

### Patients and samples

Peripheral blood (PB) serum samples from MM patients and healthy donors (HD) were obtained by the Institute of Hematology and Blood Disease Hospital for this study as previously described [15]. The myeloma patients included in this study were from a prospective, nonrandomized clinical trial (BDH 2008/02) [36]. The trial was done in accordance with the Declaration of Helsinki (Version 1996) and approved by the local ethics committees of institutions. We used criteria for symptomatic myeloma as defined by the International Myeloma Working Group [40]. The responses were also evaluated using the International Myeloma Working Group criteria [41]. A total of 152 serum samples including 108 newly diagnosed symptomatic MM (NDMM) patients and 44 healthy donors (HD) were enrolled in this study. Among the 108 NDMM patients, 104 of them have documented bone disease at different stage by whole body X-ray scanningwith a median follow-up time of 16.5 months upon diagnosis. According to their request, patients were assigned to either the bortezomib-based (arm A) or thalidomide-based (arm B) treatment. Arm A consisted 4 cycles of induction treatment with BCD (bortezomib, cyclophosphamide, dexamethasone) or PAD (bortezomib, adriamycin, dexamethasone); arm B consisted 4 cycles of induction treatment with TAD (thalidomide adriamycin, dexamethasone) or TCD (thalidomide, cyclophosphamide, dexamethasone); both arms also received the treatment with zoledronic acid every 1 to 2 months. The clinical characteristics of patients and HDs were shown in Table 1. There was no significant difference in clinical characteristics between those groups.

### Myeloma bone disease evaluation

All patients underwent modified skeletal survey including X-ray of skull, limb bone, ribs, dorsolumbar spine and pelvis within 1 week after diagnosis and radiographic examinations were obtained before the start of treatment. X-ray image patterns were defined as described previously [8–10]. Briefly, according to X-ray evaluation of the patients, bone involvement was graded to five groups: normal (grade 0); diffuse osteoporosis (grade 1); minimal lytic changes (lytic changes in one of the skeletal survey locations as mentioned above, grade 2); extensive lytic changes without pathologic fractures (lytic changes in more than one of the skeletal survey locations, grade 3); minimal or extensive lytic changes but with pathologic fractures (grade 4).

### MiRNA/RNA extraction

Total RNA including miRNAs was extracted from all serum samples using miRNeasy mini kits (Qiagen, Valencia, CA) according to the manufacturers' recommendations. Total RNA concentration was measured by using a NanoDrop ND-1000 Spectrophotometer (Thermo Scientific, Wilmington, DE). The purity of RNA samples was determined by a 260/280 ratio.

### MiRNA profiling and data analysis

To study the differential miRNA expression in MM patients, we performed miRNA expression profiling of the serum samples of 7 newly diagnosed symptomatic MM patients and 5 healthy donors using the miRCURY^™^ LNA Array (version 11.0, Exigon, Denmark) system. The microarray for miRNA profiling was conducted by the China Shanghai Kangcheng Technology Co, Ltd [16]. Briefly, 3 μg RNA samples were labeled with the Exiqon miRCURY Hy3/Hy5 power labeling kit and hybridized to the miRCURY LNA Array station. Scanning was performed using the Axon GenePix 4000B microarray scanner. GenePix pro version 6.0 was used to read raw image intensity. The intensity of the signal was calculated after background subtraction, and replicated spots on the same slide were averaged to obtain median intensity. The median normalization method was used to acquire normalized data (foreground minus background divided by median). The median was the 50 th percentile of miRNA intensity and was > 50 in all samples after background correction. The threshold value for significance used to define up-regulation or down-regulation of miRNAs was a fold change > 1.5, with *p* < 0.05 calculated by a student's *t* test.

### Candidate miRNA level confirmation by RT-qPCR

Individual miRNA assays for 4 miRNAs (hsa-miR-214, hsa-miR-135b, hsa-miR-132 and hsa-miR-92a) were performed using the All-in-One^™^ miRNA First-strand cDNA synthesis kit and miRNA RT-qPCR detection kit (GeneCopoeia, China). Quantitative PCR for miRNA was carried out under the following conditions: 95°C for 5 min, 30–50 cycles of 95°C for 5 s and 60°C for 40–60 s depending on different miRNA study followed by a final dissociation analysis. MiRNA expression for each sample was normalized to the internal control miR-423–5p which is stably expressed in serum of MM patients and calculated with 2^−ΔΔct^ [15], with three biological replicates of comparative RT-qPCR.

### Statistical analysis

Data was analyzed using SPSS version 17.0 (IBM, Chicago, IL) with the Youden's Index used to identify optimal cut-off points. Means of two groups were compared using student's *t* test. Means of more than two groups were compared using one-way ANOVA followed by multiple comparisons with Tukey method. PFS was calculated as from the initiation of therapy to progression, date of death or the last follow up. OS was measured as from the initiation of treatment to the date of death or last follow up according to the international uniform response criteria [25]. The survival curves were plotted using the Kaplan-Meier method, with differences assessed by the log-rank test. The correlation coefficients (r) were calculated by using the Spearman correlation. *P* values < 0.05 were considered to be significant for all analyses.

## SUPPLEMENTARY MATERIALS FIGURE


