# Supplementary material for: Serum high expression of miR-214 and miR-135b as novel predictor for myeloma bone disease development and prognosis
Source: Oncotarget. 2016 Feb 11;7(15):19589–600. doi: 10.18632/oncotarget.7319 (PMC4991403; doi:10.18632/oncotarget.7319)
Supplement: Supplementary file 1 [file oncotarget-07-19589-s001.pdf]

Serum high expression of miR-214 and miR-135b as novel predictor for myeloma bone disease development and prognosis

Supplementary Materials

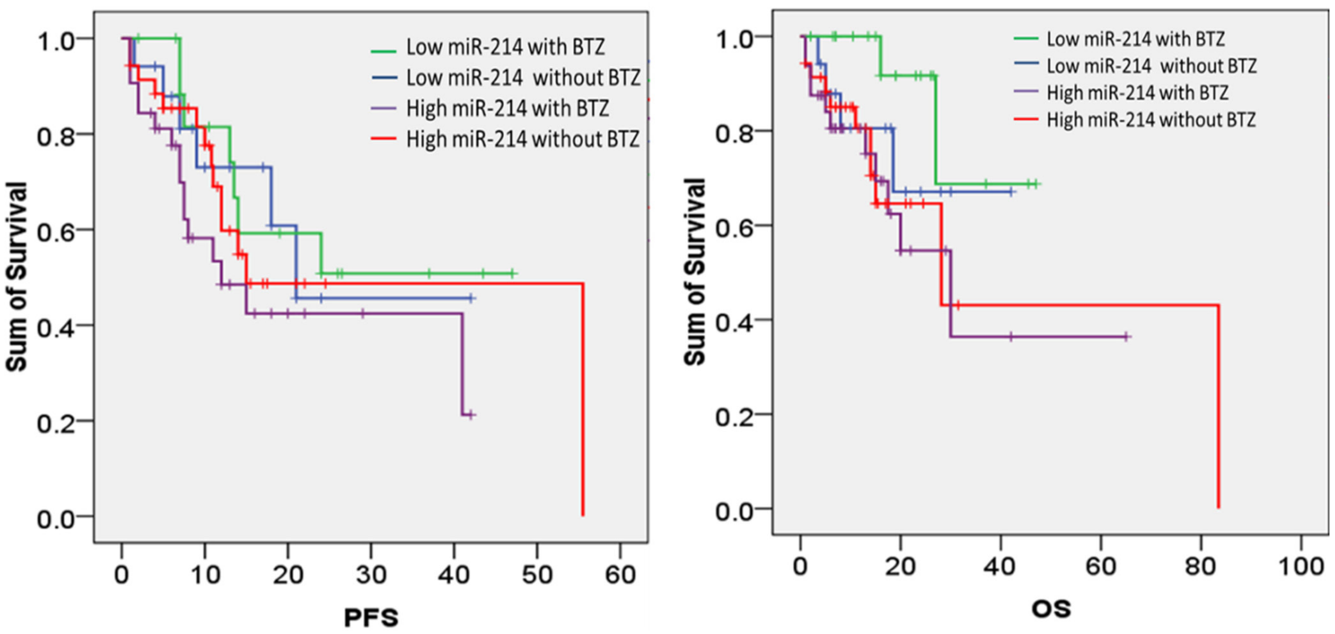

Supplementary Figure S1: Bortezomib-based therapy did not extend survival of patients with high level of miR-214. PFS and OS were evaluated for MM patients having high level of serum miR-214 with or without bortezomib treatment. Survival analysis was carried out using Kaplan–Meier survival analysis, with the differences between curves analyzed via a log-rank test. Significance was defined as $p < 0.05$.
